# Outbreak-associated *Salmonella enterica* Serotypes and Food Commodities, United States, 1998–2008

**DOI:** 10.3201/eid1908.121511

**Published:** 2013-08

**Authors:** Brendan R. Jackson, Patricia M. Griffin, Dana Cole, Kelly A. Walsh, Shua J. Chai

**Affiliations:** Centers for Disease Control and Prevention, Atlanta, Georgia, USA

**Keywords:** Salmonella enterica, bacteria, enteric infections, serotypes, foodborne diseases, food commodities, outbreaks, United States

## Abstract

*Salmonella enterica* infections are transmitted not only by animal-derived foods but also by vegetables, fruits, and other plant products. To clarify links between *Salmonella* serotypes and specific foods, we examined the diversity and predominance of food commodities implicated in outbreaks of salmonellosis during 1998–2008. More than 80% of outbreaks caused by serotypes Enteritidis, Heidelberg, and Hadar were attributed to eggs or poultry, whereas >50% of outbreaks caused by serotypes Javiana, Litchfield, Mbandaka, Muenchen, Poona, and Senftenberg were attributed to plant commodities. Serotypes Typhimurium and Newport were associated with a wide variety of food commodities. Knowledge about these associations can help guide outbreak investigations and control measures.

*Salmonella enterica* is estimated to cause 1.2 million illnesses each year in the United States and to be the leading cause of hospitalizations and deaths from foodborne disease (*1*). Because of the major public health role of *Salmonella* infections, the US Department of Health and Human Services has made decreasing the nationwide incidence of these infections by 25% a Healthy People 2020 national goal (*2*). Overall, salmonellosis incidence has not decreased in the past decade; the incidence has substantially increased for some serotypes and decreased for others (*2**,**3*). Focused attention on determining sources of *Salmonella* infections will be vital to reach the 25% target reduction in these infections.

*Salmonella* serotypes differ in their natural reservoirs and ability to cause human infections (*4**– **6*); only a small proportion of >2,500 serotypes cause most human infections (*4**,**7*). In 2009, only 20 serotypes comprised >82% of the ≈36,000 serotyped human-derived *Salmonella* isolates in the United States that were reported to the Centers for Disease Control and Prevention (*3*). A few serotypes have been associated with specific animal reservoirs. For example, serotype Dublin, which caused 103 laboratory-confirmed human infections in 2009 (*3*), is found predominantly in cattle (*5*). However, reservoir sampling alone has limited use in predicting the contribution of a reservoir to the incidence of human illness (*8*).

Outbreak data and case–control studies have linked some serotypes to certain foods or exposures (e.g., serotype Enteritidis to eggs and chicken) (*9**–**11*). Information obtained during outbreak investigations is a key tool in understanding which foods are common sources of pathogens contributing to foodborne infections. During outbreak investigations, illnesses can be linked to a particular food by using epidemiologic or laboratory evidence (*12*). To our knowledge, no systematic examination of *Salmonella* serotypes and food vehicles implicated in outbreaks has been reported. We analyzed foodborne disease outbreak data to determine associations between food commodities and serotypes to help inform future outbreak investigations, foodborne illness source attribution analyses, and control measures.

## Methods

State, local, and territorial health departments voluntarily submit reports of foodborne disease outbreak investigations to the Foodborne Disease Outbreak Surveillance System (FDOSS) of the Centers for Disease Control and Prevention. A foodborne disease outbreak is defined as >2 cases of a similar illness resulting from ingestion of a common food. Submitted reports include a description of the pathogen, the implicated food(s), the main ingredients of the food, and the contaminated ingredient, if known (*13*). When a *Salmonella* sp. is the etiologic agent, public health laboratories serotype the isolate. A *Salmonella* sp. is considered the confirmed etiology of an outbreak when the same serotype is isolated from >2 ill persons or when the bacterium is isolated from an epidemiologically implicated food (*13*).

To standardize the analysis of foods, we used a modified version of an existing classification scheme (*14*) to categorize reported foods into 1 of 20 mutually exclusive food commodities. Foods were classified into a single food commodity if a single ingredient was implicated or if all ingredients in a food belonged to a single food commodity. We then combined the individual food commodities into 3 broad food commodity groups: 1) aquatic animal–derived food commodities (crustaceans, fish, and mollusks); 2) land animal–derived food commodities (dairy, eggs, beef, game, pork, chicken, turkey, and duck); and 3) plant-derived food commodities (grains–beans, oils–sugars, fruit, nuts, fungi, sprouts, leafy vegetables, root vegetables, and vine–stalk vegetables).

We reviewed all reports of foodborne outbreaks of *Salmonella* infections to FDOSS during 1998–2008 and included in the analysis those outbreaks caused by a single, laboratory-confirmed serotype. We excluded outbreaks in which multiple etiologies were reported, that had an unknown serotype, or that could not be assigned to 1 of the 20 food commodities.

Among all salmonellosis outbreaks and for each *Salmonella* serotype, we calculated the frequency and percentage of outbreaks associated with each food commodity. For each serotype, we also determined the percentage of outbreaks associated with animal-derived food commodities (land and aquatic) and plant-derived food commodities. We calculated the Gini coefficient as a descriptive measure of the magnitude of food commodity diversity, or inequality (*15*) among outbreaks caused by a particular serotype. The Gini coefficient was chosen as a measure of diversity because it provides an easily interpretable range of values from 0 to 1. A Gini coefficient of 0 indicates an equal distribution of outbreaks caused by a serotype across all food commodities, and a value of 1 indicates that all outbreaks were attributed to a single food commodity.

## Results

During 1998–2008, a total of 1,491 outbreaks of *Salmonella* infections were reported to FDOSS, and 1,193 (80%) were caused by a single serotype. Of the single-serotype outbreaks, 595 (50%) had an implicated food, and 403 (34%) could be assigned to a single food commodity. Among these 403 outbreaks, 47 serotypes were reported; 23 serotypes caused ≥3 outbreaks. Of the 47 serotypes reported, the 4 most common caused 66% of the 403 outbreaks (Enteritidis 144 [36%], Typhimurium 58 [14%], Newport 40 [10%], and Heidelberg 24 [6%]). Overall, eggs were the most commonly implicated food commodity (112 outbreaks, 28%), followed by chicken (64 outbreaks, 16%), pork (37 outbreaks, 9%), beef (33 outbreaks, 8%), fruit (33 outbreaks, 8%), and turkey (28 outbreaks, 7%) (Table 1, Appendix).

**Table 1:** Percentage of *Salmonella enterica* serotypes attributed to specific food commodities for 403 outbreaks, Foodborne Disease Outbreak Surveillance System, Unites States, 1998–2008*

Serotype (no. outbreaks)	Commodity															
	Eggs	Chicken	Pork	Beef	Fruit	Turkey	Vine–stalk veg.	Sprouts	Dairy	Aquatic animals†	Leafy veg.	Game	Nuts	Grains–beans	Root veg.	Other‡
Enteritidis (144)	65	13	1	4	3	5	1	3	0	3	1	0	1	0	0	0
Typhimurium (58)	7	26	14	10	0	7	5	3	16	2	2	3	2	0	2	2
Newport (40)	0	13	10	15	15	8	15	0	10	0	8	3	0	0	5	0
Heidelberg (24)	42	33	0	4	4	17	0	0	0	0	0	0	0	0	0	0
Braenderup (10)	10	30	10	0	0	0	30	20	0	0	0	0	0	0	0	0
Javiana (10)	0	10	20	0	30	0	20	0	0	0	20	0	0	0	0	0
Saintpaul (9)	11	0	0	11	11	33	11	22	0	0	0	0	0	0	0	0
Hadar (8)	13	38	13	0	0	38	0	0	0	0	0	0	0	0	0	0
Infantis (7)	0	0	57	29	0	0	0	0	0	0	0	0	0	14	0	0
Montevideo (7)	0	0	29	14	0	14	0	14	29	0	0	0	0	0	0	0
Thompson (6)	0	17	0	33	0	0	17	0	0	0	17	0	17	0	0	0
Agona (5)	0	20	0	0	0	20	0	0	0	0	0	20	0	40	0	0
Litchfield (5)	0	0	0	0	100	0	0	0	0	0	0	0	0	0	0	0
Muenchen (5)	0	20	0	0	40	0	0	40	0	0	0	0	0	0	0	0
Anatum (4)	0	0	25	25	25	0	0	0	0	0	0	0	0	0	0	25
Berta (4)	0	0	25	25	25	0	25	0	0	0	0	0	0	0	0	0
I 4,[5],12:i:- (4)	25	75	0	0	0	0	0	0	0	0	0	0	0	0	0	0
Mbandaka (4)	0	0	25	0	0	0	0	75	0	0	0	0	0	0	0	0
Oranienburg (4)	0	25	0	25	50	0	0	0	0	0	0	0	0	0	0	0
Poona (4)	0	0	0	0	100	0	0	0	0	0	0	0	0	0	0	0
Uganda (4)	0	0	100	0	0	0	0	0	0	0	0	0	0	0	0	0
Senftenberg (3)	0	0	0	33	33	0	0	0	0	0	33	0	0	0	0	0
Weltevreden (3)	0	0	33	0	0	0	0	0	0	67	0	0	0	0	0	0
Other (31)	3	13	16	13	6	6	6	10	3	10	3	6	3	0	0	0
Total (403)	112	64	37	33	33	28	21	19	16	11	10	6	5	3	3	2

*veg, vegetables. †Crustaceans, fish, and mollusks. ‡Duck (serotype Anatum) and fungus (serotype Typhimurium).

The most commonly implicated food commodity differed by *Salmonella* serotype (Table 1). Eggs were the most common food commodity for outbreaks caused by serotypes Enteritidis (93 [65%] of 144 outbreaks) and Heidelberg (10 [42%] of 24 outbreaks). Egg-associated serotype Enteritidis outbreaks accounted for 23% of all single food commodity outbreaks. Chicken was the most common food commodity for serotypes I 4,[5],12:i:- (3 [75%] of 4 outbreaks) and Typhimurium (15 [26%] of 58 outbreaks). Pork was the most common food commodity for serotypes Uganda (all 4 outbreaks) and Infantis (4 [57%] of 7 outbreaks). Fruit was the most common food commodity for serotypes Litchfield (all 5 outbreaks), Poona (all 4 outbreaks), Oranienburg (2 [50%] of 4 outbreaks), and Javiana (3 [30%] of 10 outbreaks). Turkey was the most common food commodity for serotypes Hadar (3 [38%] of 8 outbreaks) and Saintpaul (3 [33%] of 9 outbreaks). Sprouts were the most common food commodity for serotype Mbandaka (3 [75%] of 4 outbreaks). Food commodities in the aquatic animal group were the most common for serotype Weltevreden (2 [67%] of 3 outbreaks). Animal-derived food commodities were implicated in >90% of outbreaks caused by serotypes Enteritidis, Heidelberg, Hadar, I 4,[5],12:i:-, Uganda, and Weltevreden, whereas plant-derived food commodities were implicated in >50% of outbreaks caused by serotypes Javiana, Litchfield, Mbandaka, Muenchen, Poona, and Senftenberg.

Evaluation of the serotype diversity within food commodity categories (Table 2, Appendix) showed that the 112 egg-associated outbreaks were predominantly caused by *Salmonella* serotypes Enteritidis (83%) and Heidelberg (9%). Of the 64 chicken-associated outbreaks, 64% were caused by serotypes Enteritidis (28%), Typhimurium (23%), and Heidelberg (13%) combined. Among the 37 pork-associated outbreaks, serotypes Typhimurium (22%), Infantis (11%), Newport (11%), and Uganda (11%) were the most common etiology. The most common serotypes causing beef-associated outbreaks were Enteritidis (18%), Newport (18%), and Typhimurium (18%). Of the 33 fruit-associated outbreaks, 57% were caused by serotypes Newport (18%), Litchfield (15%), Enteritidis (12%), and Poona (12%) combined. Among the fruit-associated outbreaks, 17 (52%) were attributed to melons. The most common serotypes causing melon-associated outbreaks were Litchfield (29%), Poona (24%), Newport (18%), and Javiana (12%). Of the 28 turkey-associated outbreaks, 53% were caused by serotypes Enteritidis (25%), Heidelberg (14%), and Typhimurium (14%) combined. Of the 21 vine-stalk vegetable-associated outbreaks, the most common serotypes were Newport (29%), Braenderup (14%), and Typhimurium (14%). Among the vine-stalk vegetable outbreaks, 19 (90%) were attributed to tomatoes. The most common serotypes causing tomato-associated outbreaks were Newport (32%), Typhimurium (16%), Braenderup (11%), Enteritidis (11%), and Javiana (11%). Of the 16 dairy-associated outbreaks, most were caused by serotypes Typhimurium (56%) and Newport (25%). Eleven outbreaks were associated with aquatic animal–derived food commodities, of which 5 (45%) were caused by serotype Enteritidis. Of the 10 leafy vegetable-associated outbreaks, 50% were caused by serotypes Newport (30%) and Javiana (20%).

**Table 2:** Percentage of food commodity–associated outbreaks caused by specific *Salmonella enterica* serotypes for 403 outbreaks, Foodborne Disease Outbreak Surveillance System, United States, 1998–2008*

Commodity (no. outbreaks)	Serotype																							
	Enteritidis	Typhimurium	Newport	Heidelberg	Braenderup	Javiana	Saintpaul	Hadar	Infantis	Montevideo	Thompson	Agona	Litchfield	Muenchen	Anatum	Berta	I 4,[5],12:i:-	Mbandaka	Oranienburg	Poona	Uganda	Senftenberg	Weltevreden	Other
Eggs (112)	83	4	0	9	1	0	1	1	0	0	0	0	0	0	0	0	1	0	0	0	0	0	0	1
Chicken (64)	28	23	8	13	5	2	0	5	0	0	2	2	0	2	0	0	5	0	2	0	0	0	0	6
Pork (37)	5	22	11	0	3	5	0	3	11	5	0	0	0	0	3	3	0	3	0	0	11	0	3	14
Beef (33)	18	18	18	3	0	0	3	0	6	3	6	0	0	0	3	3	0	0	3	0	0	3	0	12
Fruit (33)	12	0	18	3	0	9	3	0	0	0	0	0	15	6	3	3	0	0	6	12	0	3	0	6
Turkey (28)	25	14	11	14	0	0	11	11	0	4	0	4	0	0	0	0	0	0	0	0	0	0	0	7
Vine–stalk veg. (21)	10	14	29	0	14	10	5	0	0	0	5	0	0	0	0	5	0	0	0	0	0	0	0	10
Sprouts (19)	21	11	0	0	11	0	11	0	0	5	0	0	0	11	0	0	0	16	0	0	0	0	0	16
Dairy (16)	0	56	25	0	0	0	0	0	0	13	0	0	0	0	0	0	0	0	0	0	0	0	0	6
Aquatic animals† (11)	45	9	0	0	0	0	0	0	0	0	0	0	0	0	0	0	0	0	0	0	0	0	18	27
Leafy veg. (10)	10	10	30	0	0	20	0	0	0	0	10	0	0	0	0	0	0	0	0	0	0	10	0	10
Game (6)	0	33	17	0	0	0	0	0	0	0	0	17	0	0	0	0	0	0	0	0	0	0	0	33
Nuts (5)	40	20	0	0	0	0	0	0	0	0	20	0	0	0	0	0	0	0	0	0	0	0	0	20
Grains, beans (3)	0	0	0	0	0	0	0	0	33	0	0	67	0	0	0	0	0	0	0	0	0	0	0	0
Root veg. (3)	0	33	67	0	0	0	0	0	0	0	0	0	0	0	0	0	0	0	0	0	0	0	0	0
Other‡ (2)	0	50	0	0	0	0	0	0	0	0	0	0	0	0	50	0	0	0	0	0	0	0	0	0
Total (403)	144	58	40	24	10	10	9	8	7	7	6	5	5	5	4	4	4	4	4	4	4	3	3	31

*veg., vegetables. †Crustaceans, fish, and mollusks. ‡Duck (serotype Anatum) and fungus (serotype Typhimurium).

Some serotypes were associated with a narrow range of food commodities. Among the 10 serotypes causing the most outbreaks in our study, *Salmonella* serotypes Enteritidis, Hadar, Heidelberg, and Infantis had the lowest diversity, or highest inequality (Gini coefficient ≥0.8), of implicated food commodities (Figure). Outbreaks caused by serotypes Enteritidis, Hadar, and Heidelberg were mostly attributed to eggs and poultry, and serotype Infantis outbreaks were mostly linked to pork. Serotypes Newport and Typhimurium had the greatest diversity (Gini coefficient <0.6), which reflected a wide range of implicated food commodities. Serotypes Braenderup, Javiana, Montevideo, and Saintpaul had modest diversity. Among them, serotype Montevideo outbreaks were mostly attributed to animal–derived food commodities (>80%); 30%–56% of outbreaks caused by serotypes Braenderup, Javiana, and Saintpaul were attributed to animal-derived food commodities.

**Figure:**
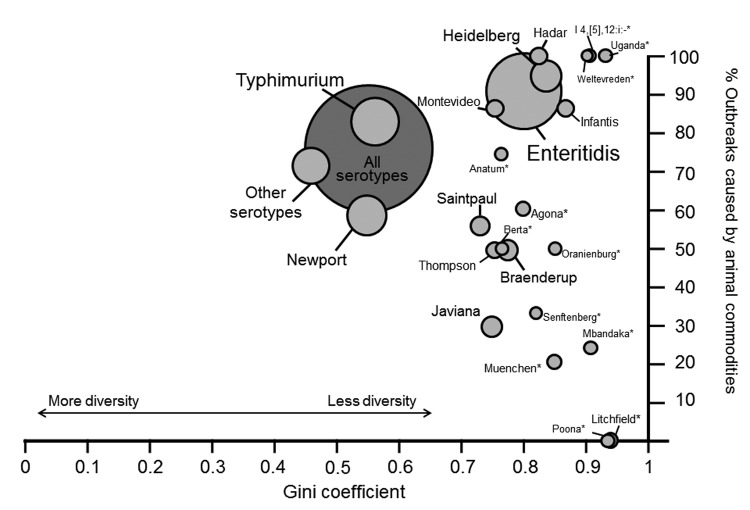
Gini coefficient and percentage of outbreaks attributed to animal commodities for each *Salmonella enterica* serotype, Foodborne Disease Outbreak Surveillance System, United States, 1998–2008. Size of circle indicates number of outbreaks for each serotype. Animal commodities include land animals (beef, chicken, eggs, game, pork, and turkey) and aquatic animals (crustaceans, fish, and mollusks). *Serotypes with <5 outbreaks. The Gini coefficient is a measure of diversity; a value of 0 indicates an equal distribution of outbreaks caused by a serotype across all commodities, and a value of 1 indicates that all outbreaks were attributed to a single commodity.

## Discussion

We found notable relationships between *Salmonella* serotypes and food commodities that point to major food reservoirs for different serotypes. Certain serotypes, in particular Enteritidis, Heidelberg, Hadar, and Infantis, caused outbreaks predominantly attributed to specific animal-derived food commodities, a finding that is consistent with results from animal reservoir sampling (*6*). We also identified serotypes that commonly caused outbreaks associated with plant-derived food commodities, particularly the fruit, vine–stalk vegetable, sprouts, and leafy vegetable food commodities. These serotypes that cause plant-associated outbreaks are found relatively infrequently in *Salmonella* reservoir studies of livestock (*6*), which suggests that serotypes with non-livestock reservoirs (e.g., environmental, amphibian, or reptile reservoirs) may be more likely to cause outbreaks by plant-based food vehicles. For example, during an outbreak investigation of serotype Poona infections attributed to cantaloupe consumption, investigators suspected that melons might have been indirectly contaminated through packing equipment or wash water contaminated by reptiles (*16*). Our findings regarding plant-associated serotypes are particularly relevant given recent increases in *Salmonella* outbreaks attributed to fruits or vegetables and a concurrent increase in infections caused by serotype Javiana (*3**,**17*), a serotype that compared with other common serotypes in this study, caused a higher percentage of plant-derived food commodity–associated outbreaks.

Our findings of predominant animal-derived food commodities for specific serotypes are supported not only by animal reservoir studies, but also by case–control studies of sporadic illness. Although the percentage of outbreaks attributed to a specific food commodity is not directly comparable to the population–attributable fraction estimated in case–control studies because the units of measure (outbreaks versus illnesses) and the method of estimating the sources of illnesses are different, our results and those of case–control studies show similar dominant food commodity reservoirs for some serotypes. For example, serotype Enteritidis was responsible for a high (83%) proportion of egg-associated outbreaks and ≈25% of chicken and turkey outbreaks; these findings are supported by case–control studies that found eggs and poultry to be common sources of serotype Enteritidis infection (*10**,**11*).

The high percentages of serotype Heidelberg outbreaks attributed to eggs, chicken, and turkey are also supported by findings from case–control studies and previous reviews (*18**,**19*). These findings suggest that these products are common vehicles for this serotype. The link we found between serotype Hadar and turkey is consistent with historical data and animal surveillance data showing that serotype Hadar is now the most common serotype isolated from turkey (*6*). The link we found between serotype Infantis and pork is also consistent with animal surveillance data showing that this serotype is commonly isolated from swine but not poultry (*6*). Three of the 4 serotypes with the lowest food commodity diversity measured by the Gini coefficient (Enteritidis, Heidelberg, and Hadar) were predominantly associated with eggs and poultry, suggesting that these serotypes are well adapted to poultry reservoirs and are a well-defined target for control measures.

Two of the most common *Salmonella* serotypes, Typhimurium and Newport, had a wider range of implicated food commodities. Serotype Typhimurium has a well-characterized ability to infect various species (*20*) and can survive for a long time in the environment (*21*); these 2 factors enhance the ability of this serotype to be one of the most common causes of salmonellosis in the United States (*2*). Although we found serotype Typhimurium was associated with several animal commodities, the most common food commodity was chicken (26% of outbreaks), indicating that chicken is a major route of exposure. Among pork-associated outbreaks, Typhimurium was the most common serotype, which corroborates animal data showing that serotype Typhimurium has emerged as the predominant serotype in swine (*6*).

For *Salmonella* serotype Newport, diversity of implicated food commodities might be related to intraserotype genetic variation because several distinct clades have been identified (*22*). Antimicrobial drug resistance data might be helpful for differentiating serotype Newport infections transmitted through animal commodities versus those transmitted by plant-derived food commodities. A sporadic case–control study found associations between infection with multidrug-resistant strains of *Salmonella* serotype Newport and beef and egg consumption, whereas infection with pansusceptible strains was associated with direct or indirect exposure to frogs or lizards (*23*). In a similar manner, strains of serotype Newport causing several outbreaks attributed to beef or dairy products have been multidrug resistant (*24**,**25*), whereas outbreaks attributed to produce have generally been pansusceptible (*26**,**27*). Therefore, pansusceptibility might be a marker for serotype Newport strains with environmental reservoirs and a greater potential for transmission though produce. Our findings support the hypothesis that *Salmonella* serotypes with environmental, amphibian, or reptile reservoirs might be more likely to be transmitted by fresh produce.

All outbreaks caused by *Salmonella* serotypes Litchfield and Poona were attributed to fruit. These 2 serotypes were responsible for 25% of fruit outbreaks despite representing only 2% of outbreaks caused by all serotypes in our study. Both serotypes have been established as reptile associated (*28**,**29*) and reptiles might play a role in fruit contamination (*16*). In a similar manner, 70% of outbreaks caused by serotype Javiana, a serotype associated with reptile and amphibian contact (*30*), were linked to plant-derived food commodities.

Among *Salmonella* serotypes causing small numbers of outbreaks, several had particular animal reservoirs. This result is consistent with reported findings. For example, 2 of 3 serotype Weltevreden outbreaks were associated with aquatic animals, and serotype Weltevreden was the most common serotype found in a survey of imported seafood (*31*). Serotype Agona was responsible for 2 of the 3 outbreaks attributed to grains–beans, both traced to the same facility 10 years apart (*32*). This serotype was introduced into the United States in the 1970s by another dry food product, contaminated fishmeal used in livestock feed (*33*), which suggests good survival of this serotype in dry environments and products.

*Salmonella* serotype Agona also caused outbreaks attributed to chicken and turkey, consistent with animal surveillance data documenting its frequent isolation in swine, chicken, and turkey since its introduction in animal feed (*6**,**34*). All 4 serotype Uganda outbreaks were attributed to pork, and all 4 serotype I 4,[5],12:i:- outbreaks were linked to eggs or poultry, suggesting that these food products are reservoirs. Serotype I 4,[5],12:i:- emerged as a cause of human illness in the early 1990s and is now one of the 10 most common serotypes in humans in the United States (*35*). Serotype Senftenberg is one of the most commonly isolated serotypes from turkeys and chickens (*6*) but was the cause of only a few outbreaks (all nonpoultry) in our study, suggesting that poultry is not the only food serving as a vehicle for transmission of serotype Senftenberg to humans.

Outbreak-associated illnesses represent only a small fraction of all *Salmonella* infections (*1*), and food vehicles responsible for outbreaks might differ from those causing sporadic infections. During the 11 years of our study, changes in product contamination frequency or consumption patterns might be associated with changes in the distribution of serotypes causing illness in the general population or the proportion of sporadic illnesses associated with specific food commodities. In a recent analysis of the distribution of serotypes causing foodborne disease outbreaks (*36*), the proportion of outbreaks caused by serotype Enteritidis decreased from 44% of *Salmonella* outbreaks during 1998–2000 to 24% during 2006–2008, and the percentage of outbreaks caused by *S. enterica* remained relatively constant. That study lacked the statistical power to detect changes over time in the percentages of outbreaks associated with most serotype–food commodity pairs, but found that the percentage of outbreaks caused by *Salmonella* and eggs decreased from 33% during 1998–1999 to 15% during 2006–2008.

Although outbreak data provide one of the only direct connections between food sources and infection, outbreak investigations are frequently unable to confirm the single contaminated food vehicle, limiting our ability to detect major changes over time. In our study, <33% of outbreaks had an implicated food that could be assigned a commodity. Investigators may also report suspected food vehicles on the basis of prior knowledge of the most likely foods associated with the serotype; this reporting technique would bias results toward these typical foods. Although genetic heterogeneity and differences in reservoirs exist within serotypes (*22**,**37*), our results demonstrate that serotyping provides helpful discrimination among certain serotype–food commodity pairs. Further subtyping of *Salmonella* serotypes could help identify major subtype–food commodity relationships, particularly for common serotypes like Enteritidis and Newport.

This systematic examination of foodborne disease outbreaks by *Salmonella* serotype and implicated food commodity provides major evidence linking serotypes to likely reservoirs and pathways of food contamination. Our analysis could have used outbreak-associated illnesses rather than outbreaks; the attributed sources would have been the same, but the percentages would have differed. However, the goal of this study was to describe major commodity sources by serotype, and this goal was not greatly influenced by the number of outbreak-associated illnesses. Using outbreaks or illnesses for analysis would not provide information about the proportion of sporadic illnesses that can be attributed to specific food commodities; more complex models are needed for such analyses (*14*). The results of our analysis can provide guidance to investigators when forming hypotheses about contaminated food sources during outbreak investigations, and in suggesting the likely contaminated ingredient in outbreaks associated with foods containing ingredients from multiple commodities. Investigators should also remain alert to uncommon or novel food vehicles, which are regularly being identified (*38*). Armed with knowledge of serotype–food commodity associations, public health officials may be able to more quickly form hypotheses, identify likely sources of contamination, and prevent illnesses.
